# Development of versatile affinity‐based system for one step purification process: Case of Group A *Streptococcus* vaccine

**DOI:** 10.1002/bit.28199

**Published:** 2022-08-13

**Authors:** Anne Chevrel, Leo Candela, Elisa Innocenti, Carolin Golibrzuch, Romas Skudas, Achim Schwämmle, Manuel J. T. Carrondo, Olivier Kitten, Mikkel Nissum, Ricardo J. S. Silva

**Affiliations:** ^1^ Affilogic Nantes France; ^2^ GSK Siena Italy; ^3^ Merck Darmstadt Germany; ^4^ iBET Instituto de Biologia Experimental e Tecnológica Oeiras Portugal

**Keywords:** affinity chromatography, custom affinity ligands, manufacturing, nanofitins, purification, vaccines

## Abstract

Affinity capture is one of the most attractive strategies for simplifying downstream processing. Although it is a key mainstream approach for antibody purification, the same is not true for other biologics such as vaccines, mainly due to the lack of suitable affinity material. In this study, a novel custom affinity system is introduced permitting widespread adoption of affinity capture for the purification of biologics beyond antibodies. This is illustrated here by the development of a one‐step purification process of a mutant form of streptolysin O (SLO), a vaccine candidate against *Streptococcus pyogenes* infection. The system consists of the association of custom ligands based on the Nanofitin protein scaffold, with Eshmuno® industry‐grade chromatography medium. The Nanofitins were selected for their specificity to the target product. The newly developed affinity medium was used at different column sizes to monitor scalability from process development (1 ml) and robustness verification (5 ml) to pilot (133 ml) and technical (469 ml) runs. The single‐step affinity purification consistently delivered high purity product (above > 90%) and improved performances compared with the current three‐step process: reduced process time and footprint (3 to 1 step) and increased product yields (0.31 g vs. 0.04 g of SLO per kg of harvest broth). The custom affinity system herein described can potentially be applied to any biologic for which a specific Nanofitin is identified, thus establishing a platform with a strong impact on the manufacturing of vaccines and other biological targets.

## Graphical abstract

Novel custom purification system for affinity capture of biologics was introduced in this study. Here, Chevrel et al. show that the association of custom ligands based on Nanofitin protein scaffold with Eshmuno® industry‐grade chromatography medium enable single‐step affinity purification. Purifying a vaccine candidate against *Streptococcus pyogenes* infection, the authors have demonstrated the potential of the custom affinity medium in a cGMP scenario with significant improvements in product yield and process time.

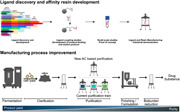

## 1 INTRODUCTION

Affinity chromatography (AC) is one of the most versatile and powerful purification tools to extract a product of interest from a crude medium. The working principle of this method is based on a reversible specific binding interaction between an immobilized ligand and the product to purify. With the proper set of binding and elution conditions, AC can provide high purity material in a single‐step purification process. As such, it bears the potential to simplify process architecture by limiting the number of purification steps, without compromising product purity and recovery. As an example, AC has been fully integrated with the downstream processes of therapeutic antibodies, creating a highly competitive space around the commercialization of engineered protein A resins (Mehta et al., 2018). Many other industrial purification processes, such as the purification of vaccines (Carvalho et al., 2021; Lightfoot & Moscariello, 2004; Plotkin et al., 2017; Yang & D'Amore, 2014; Zhao et al., 2019), could benefit from the implementation of an AC step. Despite a consensual interest from the biomanufacturers, its implementation remains very limited. The main obstacles are (i) the availability of custom ligands for each different product to purify that need to be developed in a streamlined and rapid fashion, and (ii) an effective chain to support the introduction of such AC products into an industrial scenario from establishing the ligand, scaling up, to the qualification and validation of the custom affinity material (Zhao et al., 2019).

With this goal, the European Union‐funded project—DiViNe (DiViNe Project, 2021), a consortium involving complementary partners (iBET, Affilogic, Aquaporin, Merck, GenIbet Biopharmaceuticals, and GSK) has been gathered, to build an affinity purification system that can be applied to several types of vaccines within a relevant timeframe from ligand design to current good manufacturing practice (cGMP) affinity resin. This is dubbed the “DiViNe concept.”

Nanofitins are small, single‐chain, cysteine‐free affinity proteins derived from Sac7d, a 66‐amino acids protein isolated from the hyperthermophile archaeon *Sulfolobus acidocaldarius* initially discovered in hot springs with low pH (85°C and pH 2) (Mouratou et al., 2007). The randomization of the natural DNA‐binding site of Sac7d yields libraries of Nanofitins, from which high specificity and affinity ligands to diverse molecules can be isolated in a time‐efficient manner. Nanofitins fold spontaneously into a well‐characterized OB‐fold comprising an incomplete five‐stranded β‐barrel capped by an α‐helix (Huet et al., 2015) and share the stability profile of the parent protein to wide ranges of pH, temperature, and chemicals. Their binding site is located on the opposite face to their N and C terminus ends, which can be easily modified by engineering and used for regioselective immobilization without impairing their binding activity. All those properties make Nanofitins an interesting platform for the custom generation of ligands for affinity separation (Béhar et al., 2016).

The current manuscript reports the generation of a Nanofitin‐based custom affinity resin for vaccine purification. Purification was performed up to an industrial scale to demonstrate its relevance and benefits for manufacturing by improving the time and yield of downstream processing. The target used in this study is a protein component from a candidate vaccine against *Streptococcus pyogenes*, a spherical Gram‐positive microorganism causing a wide panel of diseases in humans, ranging from mild pharyngitis to severe and invasive diseases such as necrotizing fasciitis. The protein streptolysin O (SLO) is one of the most important virulence factors of the pathogen (Bensi et al., 2012; Zhu et al., 2017). A recombinant SLO protein, expressed in *Escherichia coli*, with a double mutation has been developed, eliminating its hemolytic activity (toxicity) and keeping the capacity to confer protection against naturally occurring SLO. The purification of this recombinant protein has to deal with the removal of product‐related impurities, as well as host cell DNA and host cell proteins. Currently, the purification of SLO makes use of three orthogonal chromatographic steps. Each of these steps, with an associated product loss, are required to obtain the specified level of purity at the expense of recovery yield (Chiarot et al., 2013). The manuscript outlines the steps taken to prepare a technical‐scale affinity column under the DiViNe project concept. Additionally, the developed affinity media was evaluated for the purification of SLO from cell lysates, with the newly developed process being benchmarked against the current state‐of‐the‐art. In addition to minimizing downstream processing time and increasing recovery yield, this approach could be applied as a platform to a variety of molecules with a similar strategy, simply by changing the immobilized ligand that confers specificity to the affinity capture step.

## 2 MATERIALS AND METHODS

### 2.1 Ligand generation and selection

#### 2.1.1 Ribosome display selection rounds and isolation of clones

The combinatorial library of Nanofitins was prepared as previously described (Mouratou et al., 2007, 2012). The PCR‐amplified library was transcribed, and the selection was done at 4°C as described by Mouratou et al. (2012). The pressure of selection was adjusted by gradually increasing the time in wash steps (8 washes of 30 s, 3 min and 15 min for Rounds 1, 2, 3, respectively, and 4 washes of 15 min followed with 4 washes of 30 min for Round 4). Two different strategies of elution were evaluated in parallel at Round 4, EDTA containing buffer to detach ribosome complex or acidic buffer (pH 6) to orient selection towards pH‐dependent ligands, no difference could be observed in Nanofitin sequences collected after the analysis of isolated clones between both strategies. Then, in the following steps of protocols and results, to simplify understanding, clones resulting from each strategy were considered a unique pool of binders. Amplified DNA material from the fourth round was cloned between BamHI and HindIII restriction sites of the plasmid pQE‐30 (Qiagen) modified with the addition of the green fluorescent protein (GFP) sequence from *Aequorea coerulescens* in frame with the HindIII restriction site of the multiple cloning site, and the ligation mixture was transformed into *E. coli* DH5α LacIq strains (Invitrogen). At this stage, clones are in screening format with both a histidine tag (histag) and GFP tag. Clones selected on 2xYT‐agar plates containing 100 µg/ml ampicillin and 25 µg/ml kanamycin were inoculated into a deep‐well plate, expressed, and recovered in lysis supernatant for screening (Supporting Information).

#### 2.1.2 Enzyme‐linked immunosorbent assay (ELISA) screening

Streptavidin (66 nM, 100 μl/well; Sigma‐Aldrich) in Tris‐buffered saline (TBS) was immobilized on a Maxisorp plate (Nunc) by overnight incubation at 4°C. Each of the following steps was run at room temperature (RT), with shaking at 600 r.p.m. for incubation steps. The wells were washed three times with 300 µl of TBS, then blocked with 300 μl of 0.5% bovine serum albumin (BSA; Sigma‐Aldrich) in TBS (TBS‐BSA0.5%) for 1 h. The plates were flicked over and biotinylated SLO (100 μl, 40 nM) diluted in TBS‐BSA0.5% was allowed to attach to streptavidin for 1 h. Before all the following incubation steps, the wells were washed three times with TBS with 0.1% Tween 20. One hundred microliters of the crude *E. coli* extracts were applied to wells with and without immobilized antigen for 1 h. The revelation was then carried over by the addition of 100 µl of RGS His antibody horseradish peroxidase conjugate (Qiagen) diluted 1/4000 in TBS with 0.1% Tween 20 for 1 h, followed by the addition of 100 µl of a solution of o‐phenylenediamine dihydrochloride substrate (Sigma) in revelation buffer (0.05 M citric acid, 0.05% hydrogen peroxide). Absorbance at 450 nm was measured using an ELISA plate reader (Varioskan, Thermo Scientific).

#### 2.1.3 Sequencing and analysis

DNA from positive clones in the ELISA screen was extracted with a mini‐prep column (Promega) and sent for Sanger sequencing to GATC. Alignment of variable positions in the Nanofitin‐binding sites helps to determine shared patterns in binding sites constitutive of Nanofitin families.

### 2.2 Nanofitin expression and purification

#### 2.2.1 Subcloning into conjugation format

Nanofitins sequences were amplified by PCR on their extremities to introduce Gibson assembly compatible ends, as well as the TGT codon for the addition of the cysteine at the C terminus of the Nanofitin sequence. Gibson assembly protocol (Gibson et al., 2009) was used to insert the modified Nanofitin sequences in the pQe30 vector (Qiagen). Correct assembly was verified by Sanger sequencing (GATC).

#### 2.2.2 Expression and purification

Small‐scale Nanofitin expression and purification were carried out at different scales in the discovery and characterization process: 20 ml for small scale or 200 ml using a shaking flask, with a similar protocol. Nanofitins were expressed in *E. coli* DH5α LacIq strains.

Fed‐batch expression was performed at a 30 L scale with purification being carried out in several campaigns of 2.5 kg of biomass. A detailed description of the expression and purification protocols are reported in Supporting Information.

### 2.3 Nanofitins characterization

#### 2.3.1 Binding kinetics of Nanofitins ligands towards SLO

Binding kinetic parameters of the ligands on SLO were measured by BioLayer interferometry (BLI) on Octet RED96 system (FortéBio). Streptavidin biosensors were functionalized with anti‐GFP molecules. GFP‐fused Nanofitins were diluted at 100 µg/ml and loaded at 1 nm on prepared sensors, then biosensors were equilibrated for 60 s. Binding kinetic was then evaluated by simultaneously exposing biosensors to a concentration range of the target SLO (from 15.6 to 1000 nM). Association and dissociation steps were measured for 180 s each. All steps were performed in TBS containing 0.002% Tween 20% and 0.01% BSA. Biosensors were regenerated using three cycles of alternating wash for 10 s in Glycine 10 mM pH 2.5 and TBS. All the steps were run at 30°C with a continuous shake speed of 1000 r.p.m. For each set of measurements, one biosensor exposed to the 0 nM concentration was used as a background reference. Sensorgrams were processed using a single reference subtraction and analyzed using the Octet Data Analysis software 8.2 (FortéBio). Fitting was performed with a 1:1 binding fit model.

Additionally, optimum elution conditions of the affinity ligand were screened by BLI, using biotinylated Nanofitin‐coated Streptavidin sensors. For the preparation of the BLI sensors, Streptavidin‐coated SAX sensors were conditioned in phosphate‐buffered saline (PBS) and loaded with the biotinylated Nanofitin (30 nM in PBS) for ~120–150 s until a layer thickness of 1.5 nm was reached. Then, after washing with PBS, any residual Streptavidin was quenched by dipping the sensors in 100 mM Tris‐buffered 50 mM biotin solution, pH 8.0, for 300 s. Finally, the sensors were washed twice in 10 mM Na_2_HPO_4_, pH 7.2, 0.09% Tween 20. Nanofitin‐coated sensors were used each time by pair: a sensor in contact with diluted SLO and its reference in the same buffer to correct the occurring buffer shifts. To determine the optimum elution conditions, SLO was loaded under the same condition for all sensor pairs: 70 nM SLO in 10 mM Na_2_HPO_4_ buffer, pH 7.2, and containing 0.09% Tween 20. After 300 s of association, the sensors were placed in the elution condition. Different buffers with various pH and salt concentrations were used for elution. Good elution conditions were indicated by a rapid decrease of the layer thickness, that is, an instant dissociation of the affinity complex.

#### 2.3.2 Resistance to cleaning‐in‐place (CIP)‐like conditions

Nanofitins' concentration in PBS was adjusted to 50 µM, then a 10 times dilution was realized in NaOH concentrated at 0.01, 0.1, and 0.5 M or PBS (control), and incubated for 6 h at RT. Then, neutralization was performed by adding the molar equivalent of HCl into each sample followed by the addition of PBS to reach the final concentration of 1 µM for each condition before performing the binding experiment. ELISA plates were prepared as described above for ELISA screening before the addition of the crude cell extracts. Here, the prepared Nanofitins solutions (100 µl at 1 µM) were added to the well and incubated with or without antigens for 1 h at RT under shaking (600 r.p.m.). After washing the wells, revelation and reading were realized as described above. The percentage of the remaining binding signals were calculated based on the ELISA‐binding signals measured for the different NaOH concentrations divided by the binding signal observed for each Nanofitin in the control condition (PBS).

### 2.4 Ligand immobilization

Optimized ligand immobilization was carried out by mixing the stock solution of Nanofitins® (Supplementary Table 1) with Eshmuno® epoxy activated resin (Merck KGaA) in a ratio of 20:1 (ml/g). The reagents were kept under agitation for 4 h at a temperature of 40°C. The obtained resin was vacuum filtered followed by washing with the coupling buffer (Supporting Information: Table 1). The supernatant was recovered for analysis of the coupling efficiency. After washing, the resin was then subject to deactivation of the free epoxy groups by the addition of the deactivation solution (Supporting Information: Table 1) to a final concentration of 10% (v/v). The solution was kept under agitation overnight at RT. After this incubation period, the resin was vacuum filtered and washed with the deactivation solution. To ensure that the noncovalent bound ligand was removed from the prepared resin, two washing steps consisting of 0.1 M NaOH and 0.1 M glycine, pH 3.0, were performed, followed by thoroughly washing the resin with the storage solution (Supporting Information: Table 1) in which the resin was stored at a concentration of 50% (v/v).

### 2.5 Evaluation of affinity columns

#### 2.5.1 Generation of SLO feedstock

The broth of *E. coli* (Chiarot et al., 2013 *)* cells harvested from fermentation was collected using centrifugation. Subsequently, the cells were lysed to release the SLO protein by mechanical lysis using a homogenizer (Avestin C‐50 system, 2 cycles, 7–8 Kpsi). Afterwards, the cell lysate was clarified by centrifugation (12,000*g*, 2°C–8°C, 10 min) to remove cell debris followed by a 0.2 µm filtration step to obtain the SLO feedstock, starting material for the purification process. In the following experiments described, Harvest Broth (HB) refers to material collected from the end of fermentation, whereas feedstock is the HB that was processed by performing lysis and clarification before loading onto the column.

#### 2.5.2 Chromatography experiments

The prepared Eshmuno® Nanofitin resin was packed into columns (10 mm ID and 64 mm length for 5 ml, 26 mm ID and 200 mm length for 133 ml, and 50 mm ID and 239 mm length for 459 ml) using storage buffer (20% Ethanol and 150 mM NaCl) at a 10% compression rate. To assure a stable chromatographic bed, the obtained column was connected to the corresponding Akta chromatographic system and 25 ml/min flow rates were applied with 150 mM NaCl solution for 1 h in the downflow direction. The delta pressure of the column was <2.5 bar. Before implementation of the packed Eshmuno® Nanofitin column to the affinity separation of the SLO target molecule, the column was cleaned using 0.1 M NaOH solution for 30 min at 25 ml/min flow rate.

#### 2.5.3 Proof‐of‐concept (POC) and scale‐up experiments

The affinity purification of SLO target from the feedstock solution at pilot and technical scale was carried out in preconditioning the Eshmuno® Nanofitin column with 10 mM KPi pH 7.2 for 10 column volumes (CVs) followed by the loading of 100 ml feedstock solution on the 5 ml column, ~2.5 L feedstock solution for the 133 ml column, and 9.5 l feedstock solution for the 469 ml column applying a residence time between 1.5 and 2 min. After loading, a rinse step with the equilibration buffer was performed followed by a washing step with 10 mM Na_3_PO_4_, 750 mM NaCl, pH 6.8, to remove unbound impurities. The elution of the SLO target molecule was performed with 0.1 M Glycine buffer pH 4.0. The elution was collected in fractions, which were subjected to detailed analysis to estimate the quantity and purity of the purified SLO target molecule. After chromatographic purification, the Eshmuno® Nanofitin was cleaned with 10 CV of 0.1 M NaOH solution. After cleaning, the column was regenerated with Buffer A and filled with storage buffer for later use. The chromatographic experiments were carried out using Akta Avant 25 (Cytiva) for 1 and 5 ml columns and Akta Avant 150 (Cytiva) for 133 and 469 ml columns, provided with a UV detector (absorbance at 214–280–254 nm).

#### 2.5.4 Analytical assays

The process performance through the relevant steps was tested for the following outcome parameters: (i) antigen purity by sodium dodecyl sulfate–polyacrylamide gel electrophoresis (SDS‐PAGE; Invitrogen NuPAGE Bis‐Tris Precast Gels 4‐12%), (ii) antigen integrity by size exclusion–high‐performance liquid chromatography (SEC‐HPLC; column TSK gel 3000 SWXL, Tosoh Bioscience, Mobile phase: 0.1 M NaH_2_PO_4_, 0.1 M NaCl, pH 7, Water Alliance HPLC with PDA detector interfaced with Empower), (iii) antigen content and purity by Reversephase high‐performance liquid chromatography (RP‐HPLC) (Column: ACE 3 C4‐300, CPS. Waters Alliance with Fluorimeter interfaced with Empower software).

## 3 RESULTS AND DISCUSSION

The work herein reported describes the rationale and steps taken to move from an initial sample of the protein target up to the validation of downstream pilot and technical batches performed with the developed custom affinity resin. This section is divided into two main parts: (i) generation and selection of Nanofitin affinity ligands against the product to purify: recombinant SLO (Section 3.1), followed by the immobilization of the lead ligand to Eshmuno® beads resin and demonstration of column performances at 1 ml scale (Section 3.2); (ii) industrialization of the DiViNe concept, scale‐up of the ligand production for resin manufacturing (Section 3.3), and demonstration of performances in a real manufacturing scenario (Section 3.4).

### 3.1 Nanofitins discovery and characterization

**Figure 1:**
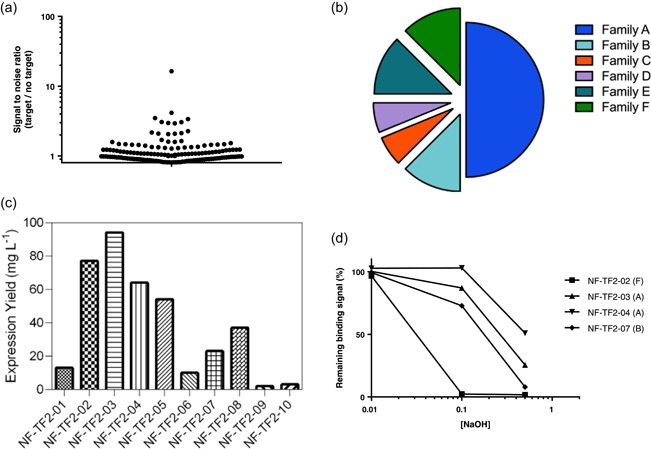
(a) Enzyme‐linked immunosorbent assay (ELISA) screening for streptolysin O (SLO) ligands. Ratio of optical density at 450 nm (binding signal) measured in the presence or absence (signal‐to‐noise ratio, S/N) of SLO were calculated for each tested clone. (b) Distribution of positive Nanofitin (S/N > 1.5) according to their sequences grouped into families based on the homology of their binding site. (c) Small‐scale expression yield (mg L^−1^) of the 10 best ligands. (d) ELISA‐binding signal recovery after accelerated cleaning‐in‐place (CIP‐like) studies: for each ligand, ratio (percentage) of optical density at 450 nm (binding signal) measured after CIP treatment compared with no CIP treatment.

To isolate affinity ligands specific to recombinant SLO, Nanofitin libraries were challenged through four rounds of ribosome display against the recombinant SLO protein used as bait. From the resulting pool of Nanofitins enriched towards binders, 180 clones were screened in crude bacterial lysis supernatant by ELISA. Twenty‐one clones presenting a favorable signal‐to‐noise ratio (>1.5) were identified, as reported in Figure 1a. All the positive clones were sequenced and then classified into six different families according to similarities between amino acid patterns within their variable domain (Figure 1b). The focus was made on 10 representatives that exhibited the highest ELISA signals while retaining sequence diversity. They were further characterized for their expression yield, binding kinetics and stability when exposed to CIP‐like conditions. All 10 ligands were expressed at a 20 ml scale in screening format (fused to a GFP tag) and purified by their histag. At this stage of the screening process, the expression system is suboptimal and values are used for clones' ranking. Ligands presenting a recovery yield below 20 mg/L were excluded from characterization (Figure 1c). The binding kinetics of the ligands were compared using BLI; all the evaluated clones exhibited affinities (*K*_D_) ranging from 10 to 100 nM (Table 1). Based on those results, the list of ligand candidates was further refined to focus only on the best clone of each sequence family (expression yield and affinity), namely NF‐TF2‐02, −03, −04, and −07.

**Table 1:** Binding kinetics measured using BLI

Ligand	*K*_D_ (nM)	*k*_on_ (M^−1^ s^−1^)	*k*_off_ (s^−1^)
NF‐TF2‐02	13.61	1.21 × 10^5^	1.65 × 10^−3^
NF‐TF2‐03	28.67	1.61 × 10^5^	4.62 × 10^−3^
NF‐TF2‐04	62.36	3.52 × 10^4^	2.20 × 10^−3^
NF‐TF2‐05	87.34	3.03 × 10^4^	2.65 × 10^−3^
NF‐TF2‐07	118.60	5.67 × 10^4^	6.72 × 10^−3^
NF‐TF2‐08	123.50	4.33 × 10^4^	5.34 × 10^−3^

Abbreviation: BLI, BioLayer interferometry.

To foster on‐resin‐oriented conjugation of the Nanofitin via thiol chemistry, subcloning was performed to remove the GFP tag and add a unique cysteine at its C terminus instead. All four cysteine‐tagged ligands could be expressed and purified with a recovery yield above 35 mg/L in a 200 ml shake flask scale (data not shown). When developing new affinity ligands, it is critical to look not only at selectivity but also at reusability. This last parameter strongly correlates with ligand stability during CIP operations. The stability of Nanofitin ligands in accelerated CIP‐like conditions was compared by a 6 h incubation in NaOH at various concentrations (0.01, 0.1, and 0.5 M). Upon neutralization, the ligands' performance was assessed by following the conserved binding activity by ELISA. Results are summarized in Figure 1d. Although incubation in 0.01 M NaOH did not impair the binding signal of the different Nanofitins, differential sensitivity to basic pH conditions was observed with the incubations at 0.1 and 0.5 M of NaOH. NF‐TF2‐03, presenting the best properties in terms of expression yield, affinity, and stability was selected as SLO affinity ligand to be incorporated on the chromatographic support.

### 3.2 Affinity process development

**Figure 2:**
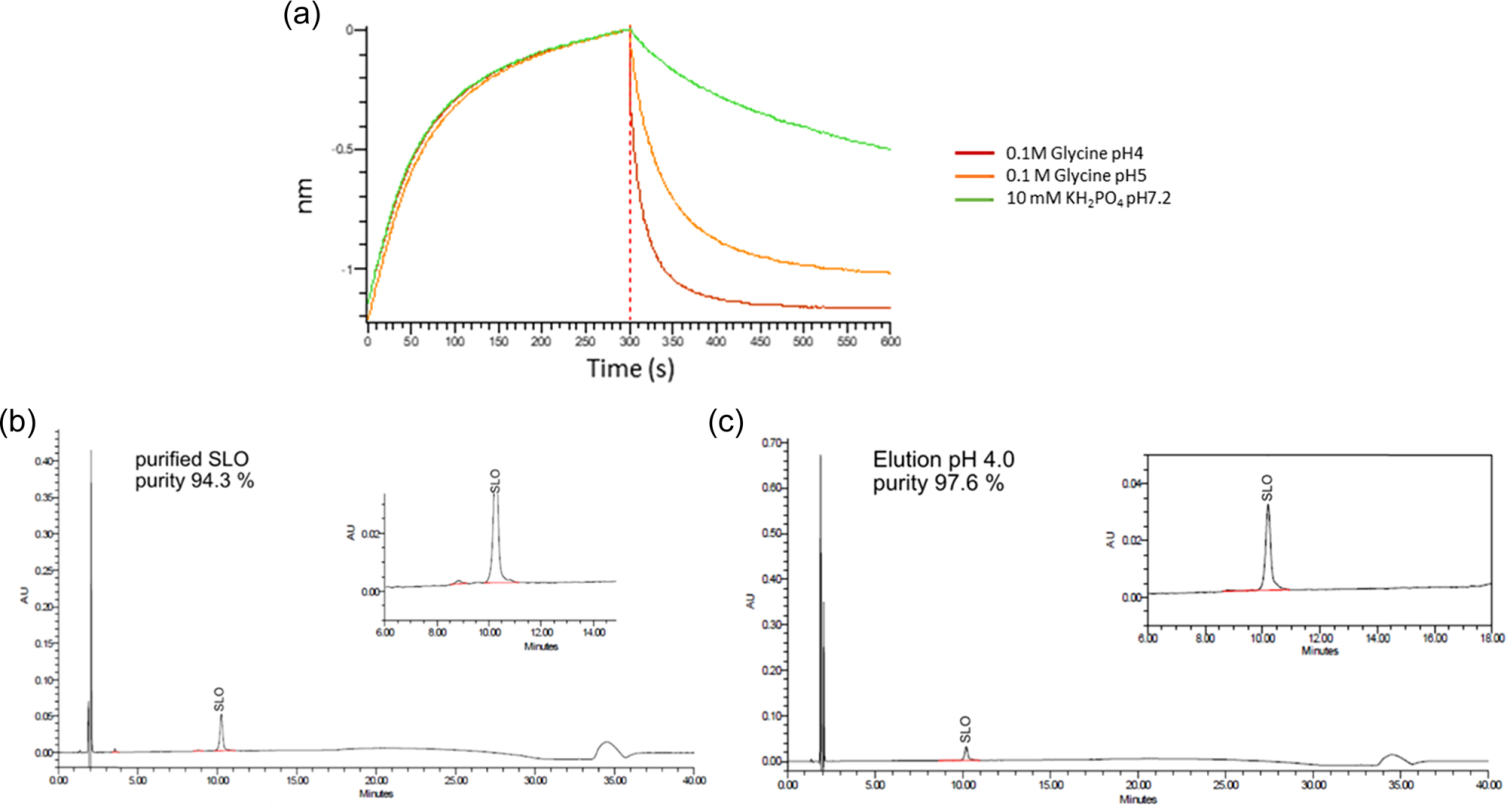
Screening of elution buffer (a) in BLI. Association (nm) of streptolysin O (SLO) on ligand modified biosensors is monitored for 300 s and then dissociation with different buffer conditions for the next 300 s. Comparison of SLO product purity by reversed‐phase high‐performance liquid chromatography (RP‐HPLC), between SLO purified by current process (b) compared with eluted SLO from affinity chromatography (AC) process established at 1 ml scale (c).

To anticipate elution conditions, release of SLO from NF‐TF2‐03 was evaluated for different buffer compositions in BLI taking into account that SLO presents reduced stability below pH 4. NF‐TF2‐03 was attached by its C‐terminal cysteine on sensors mimicking orientation on resin. Interestingly, the dissociation rate increases already at pH 5 and dissociation is complete in about 200 s in the same condition at pH 4 (Figure 2a) predicting an efficient elution once in the column. Thus, conditions for running the column were set as binding buffer: 10 mM of KH_2_PO_4_ at pH 7.2 and elution buffer 0.1 M glycine pH 4.0.

Ligand NF‐TF2‐03 was conjugated on chromatographic support and the resulting resin was characterized (Table 2). For a ligand density of 5.5 mg/ml, the affinity resin showed a static binding capacity (SBC) above 5 mg/ml of settled resin with binding efficiency of 5.3 mg of bound SLO per ml of resin, confirming the binding activity of NF‐TF2‐03 on chromatographic support and its choice as an affinity ligand.

**Table 2:** Binding and coupling efficiency of the lead Nanofitin ligand in small‐scale experiment

	resin NF‐TF2‐03
Immobilized Nanofitin (mg Nanofitin/ml of resin)	5.5
Bound SLO (mg SLO/ml of resin)	5.3

Abbreviation: SLO, streptolysin O.

NF‐TF2‐03 resin was packed into a 1 ml column to further refine the process conditions. As online detection of crude SLO by UV signal is not possible, the amount of crude feedstock that could be loaded to the column was estimated beforehand by determining the dynamic binding capacity of the column using 15 mg of pure SLO under pH and conductivity conditions corresponding to those in the crude feed. No breakthrough was visible during loading (Supporting Information: Figure 1A) and quantification of eluted fraction confirmed that all injected purified SLO was recovered, revealing a dynamic binding capacity of at least 15 mg/ml.

To further improve the purity of the resulting product, a high‐salt washing step after loading of crude SLO was added. The observed peak in the high‐salt wash could be identified as DNA impurities through UV spectroscopy (Supporting Information: Figure 4). RP‐HPLC analysis of the recovered product (Figure 2c) from the AC column reveals a purity of 97.6% when applying the additional wash and elution at pH 4.0. Retention volume and purity level appeared similar to that of the control SLO (Figure 2b).

The reusability of the new affinity medium was assessed by evaluating column capacity after multiple uses in established cleaning conditions. First, after clarified SLO material load, wash and elution, the 1 ml column was subjected to three consecutive CIP steps, each with a length of 15 CV and an increasing concentration of NaOH (0.25, 0.5, and 1 M of NaOH). Supporting Information: Figure 1B depicts the chromatogram obtained: the initial CIP step with 0.25 M of NaOH is sufficient to perform an efficient cleaning of the column, as no signal is observed in the subsequent CIP steps. Then, in the validated 0.25 M NaOH condition, column reusability was assessed by performing a cycling study using purified SLO material. As seen in Supporting Information: Figure 1C, no significant differences were observed in the elution profiles between the initial cycle and after 36 cycles, demonstrating that binding capacity is conserved, and that the affinity column can be reused at least 35 times. Elution and CIP fractions from run 36 and run 37 (mock run) were analyzed by western blotting using specific antibodies against the ligand (anti‐RGSHHH) and no ligand could be detected in any of the fractions (Supporting Information: Figure 1E. Additionally, challenging the 1 ml column with clarified SLO material originating from three different production batches, as seen in Supporting Information: Figure 1D, revealed that the implemented purification strategy is reproducible. The observed selective capture of SLO from crude material, chromatographic media capacity, and demonstrated regeneration of the column at the lab scale were a support to performing scale‐up and POC at an industrially relevant scale.

### 3.3 Towards industrial scale‐up

#### 3.3.1 Ligand expression and purification

The selected cysteine‐tagged Nanofitin–NF‐TF2‐03 was expressed at a 30 L scale. Supporting Information: Figure 2 reports the temporal profiles observed for cell growth, oxygen and glucose consumption, and the addition of base and antifoam. At the end of the fermentation, a total of 4.85 kg of biomass was recovered and processed in two purification campaigns of 2.5 kg approximately. At the end of purification, the total amount of purified Nanofitins was 16.3 g, resulting in a protein yield of 3.4 g/kg of biomass and a volumetric protein yield of 0.74 g/L. The purified Nanofitins were characterized by SDS‐PAGE and western blotting (Supporting Information: Figure 3). Nanofitins were detected in SDS‐PAGE and western blotting in the form of disulfide‐linked dimers in nonreducing conditions. In reducing conditions in the presence of dithiothreitol, the majority of the dimers were reduced and a band with a molecular weight of ~10 kDa was detected, which is in line with the expected molecular weight (~9 kDa).

#### 3.3.2 Ligand immobilization

**Figure 3:**
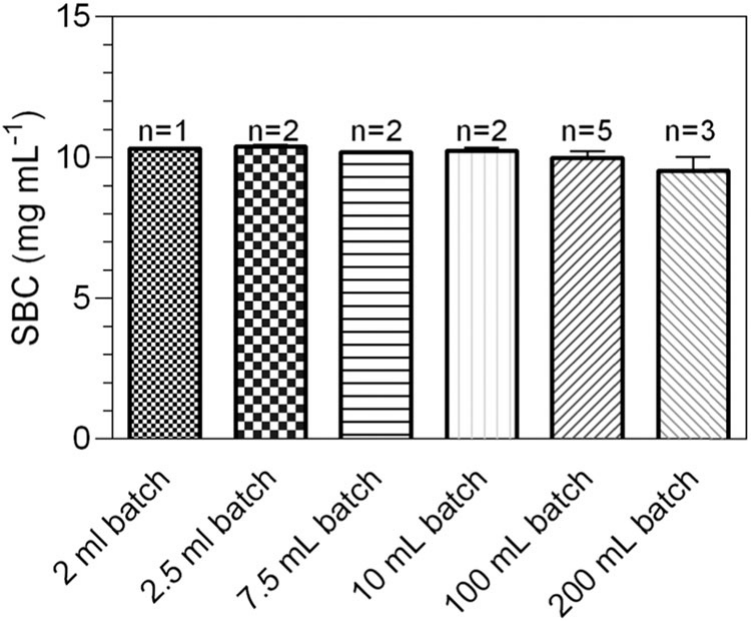
Quality control of the generated custom affinity resin. Static binding capacities (SBCs) were measured across different batches and scales.

Screening of the optimal coupling parameters was carried out in batches of 0.5 to10 ml. Parameters such as pH, reaction temperature, salt concentration, duration, Nanofitin concentration, Nanofitin/resin ratio, deactivation solution, epoxy ligand density, and reducing agent concentration were scouted to optimize the coupling efficiency. As shown in Figure 3, SBCs in the range of 10 mg of bound SLO per ml of settled resin could be obtained. The immobilization process was gradually scaled up to 200 ml batch size, monitoring the batch quality at each step by determination of the SBC (Figure 3).

A total of 800 ml of resin was thus prepared. As before, the SBC measured is in the range of 10 mg/ml across all production scales, confirming the scalability of the coupling protocol. Furthermore, the SBC obtained represents a 1.8‐fold increase when compared with the coupling studies performed during ligand discovery (Table 2).

#### 3.3.3 POC at 5 ml scale

**Figure 4:**
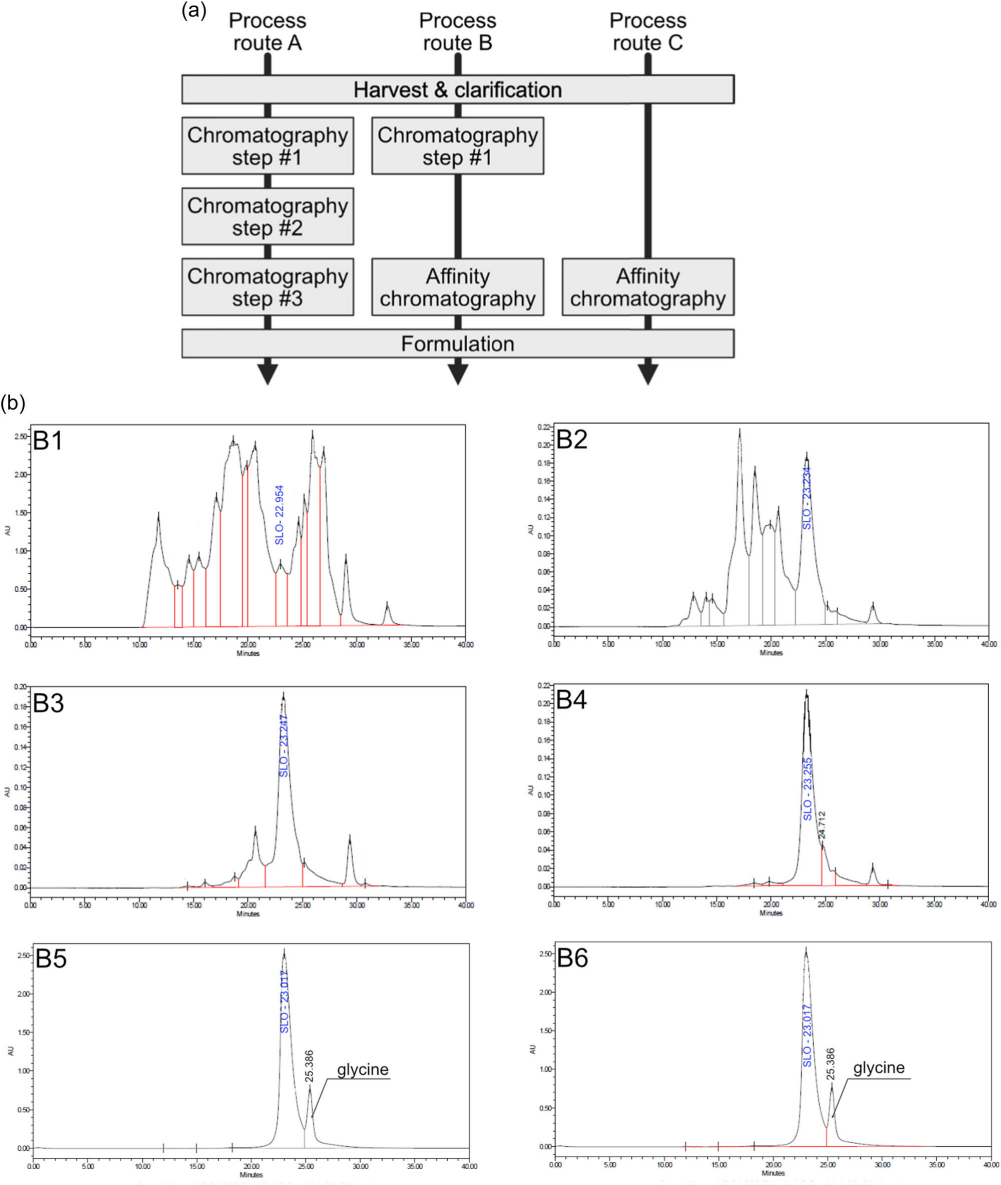
(a) Process routes. (b) Size exclusion–high‐performance liquid chromatography (SEC‐HPLC) analysis of streptolysin O (SLO) products at the different stages of the process for the three routes: SLO product after harvest and clarification (B1); for Route A: SLO product after first (B2), second (B3), and last (B4) chromatography steps; after affinity chromatography step for Route B (B5) and Route C (B6).

The potential for using the developed AC media in SLO purification process was evaluated as described in Figure 4a. Route A refers to the current downstream strategy that uses three orthogonal chromatography modes. Route B conserves the first chromatography step of the process directly associated with the AC column. Route C uses only the AC chromatography step. The performance of each step for SLO purification can be observed in Figure 4b, where chromatogram B1 corresponds to the SEC‐HPLC analysis of the clarified material (SLO monomer, purity <10%), and chromatograms B2, B3, and B4 to the product streams for each chromatography step. The measured SLO purities for each chromatography step are 28%, 69%, and 82%, respectively. The elution pool from process Routes B and C are depicted in chromatograms B5 and B6 of Figure 4b.

Table 3 reports the comparison between the full purification process using AC (*n* = 3) and the current standard purification process demonstrating high product purity for the affinity process, even slightly higher at this scale than the standard process. Furthermore, recovery is 5.25 times higher in SLO per kg of HB when compared with the standard process.

**Table 3:** SLO product properties comparison between affinity and standard process at 5 ml scale

Attributes	Affinity process (mean, *n* = 3)	Standard process
Purity RP‐HPLC (%)	94	90
Integrity SEC (%)	91	84
Purity SDS‐PAGE (%)	94	84
HCP western blotting	Negative	Negative
DNA reduction (log_10_)	4.8	2.5
DNA/protein ratio (p.p.m.)	47	29
Bioburden (plates forming units)	Negative	Negative
SLO yield (g^a^/kg HB)	0.21	0.04

Abbreviations: HB, Harvest Broth; HCP, host cell protein; RP‐HPLC, reverse‐phase high‐performance liquid chromatography; SDS‐PAGE, sodium dodecyl sulfate–polyacrylamide gel electrophoresis; SEC, size exclusion chromatography; SLO, streptolysin O.

^a^ Quantity of purified SLO determined by SEC‐HPLC.

### 3.4 Process comparison at pilot and technical scale

**Figure 5:**
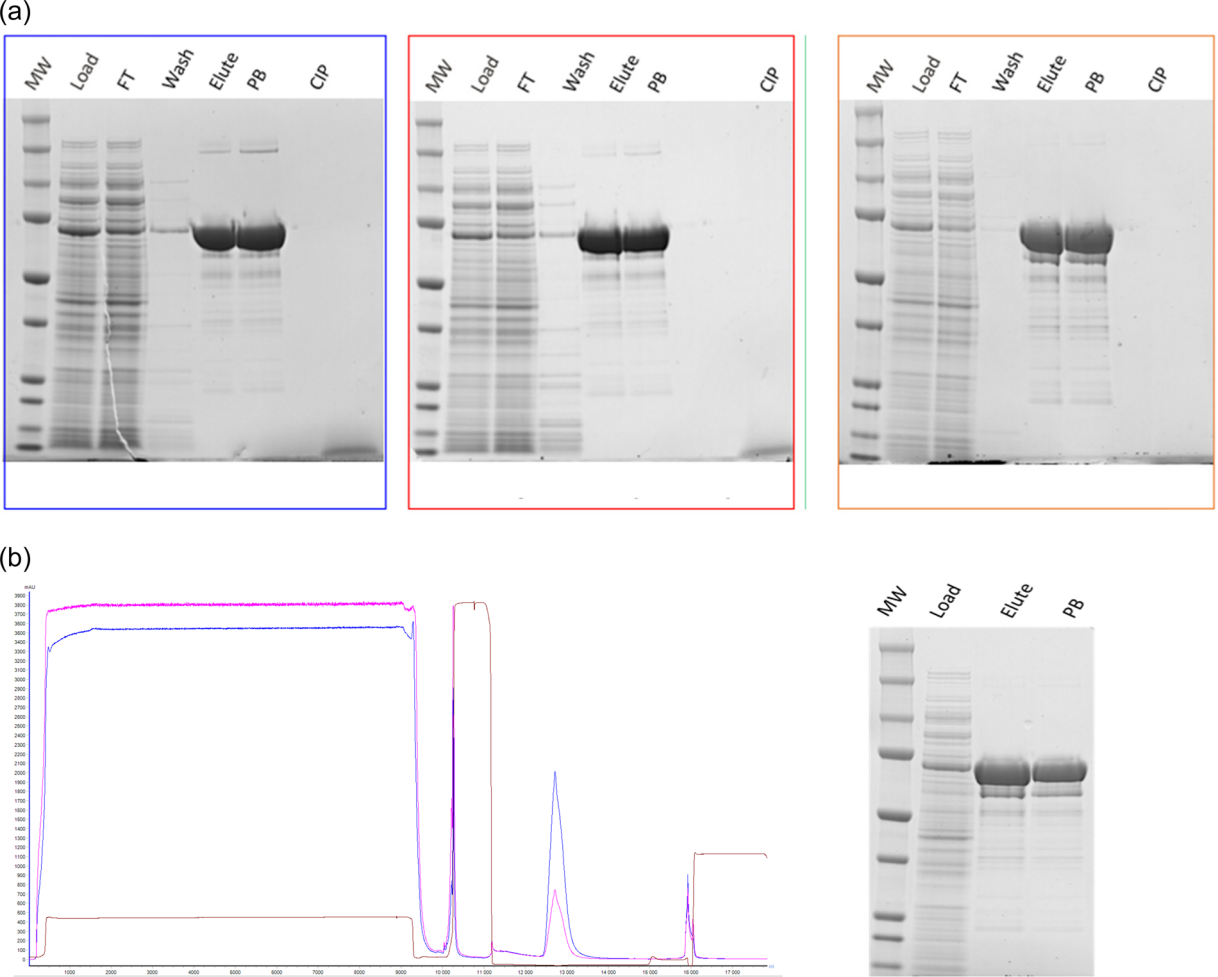
For (a), sodium dodecyl sulfate–polyacrylamide gel electrophoresis (SDS‐PAGE) analysis of chromatographic run for three different load of feedstock on the 133 ml affinity column, first (blue), second (red), and third (green) feedstock loaded: all the samples collected during the full stream runs from feed up to Purified Bulk (PB). (b) Chromatogram and SDS‐PAGE analysis of 469 ml column (blue: OD 280 nm, magenta: OD 254 nm, brown: conductivity).

Having demonstrated the potential at a 1 and 5 ml scale, the developed AC process was scaled up and compared with results obtained in POC. Three different lots of crude SLO material were processed using a packed column with 133 ml of volume to evaluate the reproducibility of the data of the Nanofitin‐based process. The three affinity runs support the reproducibility of the process consubstantiated by the SDS‐PAGE analysis reported in Figure 5a. Purity analysis by SEC‐ and RP‐HPLC of the eluted material obtained with the AC process are in the range of 89%–93% and 95%–98%, respectively (Table 4). These values are in line with those obtained by the conventional purification process and the purity values described for the 5 ml scale (Table 3). A 5 log_10_ reduction of DNA is kept when upscaling to 133 ml. Moreover, the SLO yield is maintained and even improved going from 0.21 up to 0.31 g/kg of HB at the pilot scale. At the technical scale, with 469 ml column (Figure 5b), all the quality parameters mentioned above were maintained as well as reported SLO yield.

**Table 4:** Summary of SLO product properties and yield obtained applying Route C chromatography process on different feedstock, performed with 5 ml, 133 ml, and 469 ml columns

Scale	5 ml AC column	133 ml AC column	469 ml AC column
Purity RP‐HPLC (%)	93/93/98	95/98/na	90
Integrity SEC (%)	87/93/94	89/93/91	92
DNA reduction (log_10_)	4.9/4.7/4.7	5.1/5.1/5.5	5.2
SLO yield (g^a^/kg HB)	0.16/0.23/0.25	0.36/0.27/0.31	0.36

*Note*: Results obtained with the different feedstock at the same scale are separated by a “/.”

Abbreviations: AC, affinity chromatography; HB, Harvest Broth; RP‐HPLC, reversed‐phase high‐performance liquid chromatography; SEC, size exclusion chromatography; SLO, streptolysin O.

^a^ Quantity of purified SLO determined by SEC‐HPLC.

## 4 CONCLUSIONS

The obtained results demonstrate sound applicability of the DiViNe concept as a platform for custom AC development for vaccine purification. Furthermore, strong performance throughout the development cycle of a biological product using the vaccine SLO as an example is proven. Key advantages shown include the following: (i) purity improvement from 84% to 90% in standard process versus 94% in AC process; (ii) reusability of the column over 35 regeneration cycles keeping capacity above 15 mg/ml; (iii) limited efforts were required to scale‐up the manufacturing of the custom AC medium; (iv) finally, both ligand and resin manufacturing are animal‐free and have been developed within cGMP requirements. Custom affinity column (up to 0.5 L) and the developed downstream process were assessed in a technical run within an industrial setup. Process yield has been improved more than five times with increased purity of the final SLO product. Finally, the simplified downstream process was reduced from five to three steps: harvest and clarification, AC and final polishing, and formulation delivering the final product with purity and quality requirements.

## AUTHOR CONTRIBUTIONS

*Conceptualization*: Anne Chevrel, Elisa Innocenti, Manuel J. T. Carrondo, Mikkel Nissum, and Olivier Kitten. *Methodology*: Anne Chevrel, Achim Schwämmle, Carolin Golibrzuch, and Romas Skudas. *Validation*: Anne Chevrel, Achim Schwämmle, Carolin Golibrzuch, Elisa Innocenti, Leo Candela, Manuel J. T. Carrondo, Ricardo J. S. Silva, and Romas Skudas. *Formal analysis*: Anne Chevrel, Achim Schwämmle, Carolin Golibrzuch, Elisa Innocenti, Leo Candela, Ricardo J. S. Silva, and Romas Skudas. *Investigation*: Anne Chevrel, Leo Candela, Elisa Innocenti, and Ricardo J. S. Silva. *Resources*: Anne Chevrel, Achim Schwämmle, Elisa Innocenti, and Ricardo J. S. Silva. *Writing—original draft*: Anne Chevrel, Carolin Golibrzuch, Ricardo J. S. Silva, and Romas Skudas. *Writing—review and editing*: Anne Chevrel, Achim Schwämmle, Carolin Golibrzuch, Elisa Innocenti, Manuel J. T. Carrondo, Mikkel Nissum, Olivier Kitten, Ricardo J. S. Silva, and Romas Skudas. *Visualization*: Anne Chevrel, Ricardo J. S. Silva, and Romas Skudas. *Supervision*: Anne Chevrel, Olivier Kitten, Achim Schwämmle, Mikkel Nissum, and Ricardo J. S. Silva. *Project administration*: Anne Chevrel, Achim Schwämmle, Manuel J. T. Carrondo, Mikkel Nissum, Olivier Kitten, and Romas Skudas. *Funding acquisition*: Achim Schwämmle, Olivier Kitten, Manuel J. T. Carrondo, and Mikkel Nissum.

## CONFLICT OF INTERESTS

Anne Chevrel, Leo Candela, and Olivier Kitten are employees of Affilogic. Elisa Innocenti and Mikkel Nissum are employees of GSK. Achim Schwämmle, Carolin Golibrzuch, and Romas Skudas are employee of Merck.

## Supporting information

Supplementary information.

## Data Availability

The data that support the findings of this study are available from the corresponding author upon reasonable request.
