# Supplementary material for: Development of versatile affinity‐based system for one step purification process: Case of Group A Streptococcus vaccine
Source: Biotechnol Bioeng. 2022 Aug 13;119(11):3210–20. doi: 10.1002/bit.28199 (PMC9804325; doi:10.1002/bit.28199)
Supplement: Supplementary file 1 — Supplementary information. [file BIT-119-3210-s001.docx]

# Development of versatile affinity-based system for one step purification process: case of Group A *Streptococcus* vaccine.

Anne Chevrel^1,^, Leo Candela^1^, Elisa Innocenti^2^, Carolin Golibrzuch^3^, Romas Skudas^3^, Achim Schwämmle^3^, Manuel J.T. Carrondo^4^, Olivier Kitten^1^, Mikkel Nissum^2^ and Ricardo J.S. Silva^4^

^1^Affilogic, Nantes, France

^2^GSK, Siena, Italy

^3^Merck, Darmstadt, Germany

^4^ iBET, Instituto de Biologia Experimental e Tecnológica, Oeiras, Portugal

* **Correspondence** Anne Chevrel, Affilogic, 24 rue de la Rainière, 44300 Nantes, France.

Email: [anne@affilogic.com](mailto://anne@affilogic.com)

Supplementary Information

Supplementary methods

Biotinylation of SLO

SLO biotinylation was performed by incubation of a 10 µM solution of the target protein with a 10-fold molar excess of sulfosuccinimidyl-6-(biotinamido) hexanoate (Sulfo-NHS-LC-LC-Biotin, Pierce) in 10mM KPO_4_, 80mM NaCl, pH 6.8 buffer on ice during 1 h. Excess of biotin reagent was then eliminated applying a PD-10 desalting column (Amersham 17-0851-01) equilibrated with PBS. The degree of biotinylation was determined, using the HABA-avidin assay (Sigma), as being approx. 1 molecule of biotin per protein molecule.

Nanofitins expression– 96 deep-well plate screening

96 well plates containing 0.75 mL of 2xYT medium containing 100 µg/mL ampicillin, 25 µg/mL kanamycin and 1% glucose in each well were inoculated with pîcked clones from last round of ribosome display. After overnight culture at 37 °C with shaking at 600 rpm, 0.2 mL of each culture was used to inoculate a second deep-well plate containing 1.25 mL of 2xYT medium supplemented with 100 µg/mL ampicillin, 25 µg/mL kanamycin and 0.1% glucose per well. The plate was incubated at 37 °C for 3 h with shaking at 600 rpm. Expression of the Nanofitin clones was induced by the addition of 50 µL of Isopropyl β-D-1-thiogalactopyranoside at a final concentration of 0.5 mM and incubation at 30 °C for 4 h with shaking at 600 rpm. Cells were pelleted by centrifugation (20 min at 2000g), and supernatants were discarded. Proteins were extracted with 100 µL of BugBuster® Protein Extraction Reagent (Novagen) per well with shaking for 1 h at room temperature (RT), and 350 µL of TBS (20 mM Tris-HCl, 150 mM NaCl, pH 7.4) were added. Cell debris were pelleted by centrifugation (20 minutes at 2000 x g) and supernatants were used for screening purposes.

**Nanofitins expression and purification – small scale**

Nanofitins were expressed in E. coli DH5α LacIq strains. Precultures were grown overnight at 37 °C in 2xYT medium with 1% glucose, 100 μg/mL ampicillin and 25 μg/mL kanamycin. Precultures were diluted 1:20 in 2xYT medium with 0.1% glucose, 100 μg/mL ampicillin and 25 μg/mL kanamycin and grown at 37 °C for 3 hours for small scale and to mid-log phase (OD600 = 0.8–1.0) for 200 mL shaking flask. Then, protein expression was induced by the addition of Isopropyl β-D-1-thiogalactopyranoside to the final concentration of 0.5 mM and the culture was shaken at 30 °C overnight. Bacteria were pelleted by 45 min centrifugation at 3220 x g. Cell pellets were resuspended in a pH 7.4 lysis buffer composed of 1X BugBuster Protein Extraction Reagent, 5 μg/mL DNaseI, 20 mM Tris, 500 mM NaCl, and 25 mM Imidazole. Cell lysis occurred at RT for 1 h and the suspension was centrifuged at 3220 x g for 45 min to remove cell debris. His-tagged proteins were then purified from supernatants by immobilized metal ion affinity chromatography (IMAC), using His60 Nickel Superflow resin (Takara) and a pH 7.4 elution buffer composed of 20 mM Tris, 500 mM NaCl, and 250 mM Imidazole.

Nanofitins expression and purification – fed batch

Fed-batch Nanofitin expression was performed using a 30 L Bioreactor (Bioengineering LP351). The initial batch phase consisted of a 15 L culture containing minimal medium. Initial conditions were set as follows: temperature = 37 °C, airflow = 30 Lpm, stirring = 200 RPM, and pressure = 100 mbar. Dissolved oxygen concentration was maintained above 30% saturation by operating the stirrer speed until the maximum speed was attained (1000 RPM) and then above 20% saturation by increasing pressure up to 800 mbar. The pH was kept at 7.0 by the addition of base (NH4OH, 25%) and the formation of foam was suppressed by automated addition of 20% simethicone emulsion.

Before bioreactor inoculation, a pre-inoculum culture was prepared by inoculating 100 mL minimal media, with the same composition as above except antifoam, with 1 mL cryo stock, and allowed to grow at 37 °C, 200 RPM. After 15-18 h this culture was used to inoculate 750 mL of minimal media in a 5 L vented flask with magnetic stirring. The inoculum was grown at 37 °C for 18 h and transferred to the bioreactor automatically using a controlled peristaltic pump or manually. The exponential feeding phase was started after total consumption of the initial glucose, monitored by an increase in pH (0.1 pH units above control set-point). To avoid salt precipitation in feeding solution, nutrients were fed using two separate independent feeding controls using controlled peristaltic pumps according to the equation: Q=Q0eμt, where Q is the substrate feeding rate, Q0 is the initial feeding rate (48 g/h and 4.8 g/h for feeding 1 and 2, respectively) and μ is the specific growth rate which was set to 0.15 – 0.21h-1. Cell expansion was carried until a defined feeding rate of 900 g/h (feeding 1), approx. 15 h after fed-batch start). At this point, the biomass was induced with 1 mM IPTG to start the protein expression phase. The feeding rate was then decreased by 100 g/h at each period of 1 h, to avoid substrate inhibition. The induction period lasted for 3-4 h, after which cells were harvested by centrifugation using a Beckman Avanti J-HC.

Purification was carried out in several campaigns of 2.5 kg of biomass. The biomass was resuspended in 8 L of PBS supplemented with 500 mM NaCl and 30 mM Imidazole, pH 8.0 (Buffer A), and disrupted in APV 2000 homogenizer. Cell debris were removed by centrifugation for 45 min at 4000 × g. The supernatant was filtered consecutively through 0.8 + 0.45 µm and 0.45 + 0.2 µm Sartopore Maxicap filters (Sartorius). The clarified solution was injected in a Chelating Sepharose Fast flow (GE healthcare) packed in xk50/60 columns (GE Healthcare) to a bed height of 50 cm at 10 mL/min and subsequently washed with 10 column volumes of buffer A. The bound protein was washed consecutively with 10 volumes of buffer A + 1% Triton-X100, 10 volumes of buffer A + 1 M NaCl and 10 volumes of buffer A. Nanofitins® were eluted by applying a linear gradient of 0 – 500 mM imidazole during 5 column volumes. The main peak was concentrated by tangential flow filtration 5 kDa MWCO cutoff membrane with an area of 0.1 m2 and desalted using a Sepharose G25 packed in an xk50/60 holder (GE Healthcare) equilibrated with PBS. The protein was concentrated to 50 mg/mL by tangential flow filtration (5 kDa MWCO membrane) as above and injected in a sartobind STIC nano column (Sartorius) for polishing. Finally, Nanofitins were stored at RT after filter sterilization (0.2 μm) with the addition of sodium azide at a concentration of 2 mM.

Nanofitin Biotinylation

Nanofitin was labelled with biotin via its cysteine end by incubating the PBS buffered protein (1.5 mg/mL) with a 5-fold excess of EZLink™ Maleimide-PEG2-biotin linker (ThermoFisher) for 90 min at room temperature. The excess biotinylation reagent was removed by dialysis overnight, using a cassette with a 3500 Da cutoff membrane (Slide-a-Lyzer™, ThermoFisher). The efficiency of the biotinylation was calculated 0.9 biotin molecules per molecule of protein, using the HABA/avidin assay (ThermoFisher). The protein concentration was measured by A280 absorption.

Ligand density

The amount of ligand bound to the Eshmuno® resin was determined using a bicinchoninic acid (BCA) protein assay (Thermofisher) which was carried out as follows: Reagent A (BCA) was mixed 50:1 with Reagent B (4% cupric sulfate). The resin samples were prepared by diluting 50 µL of a 50% slurry (corresponding to 25 µL of settled resin) with 450 µL milliQ water. A dilution series of Bovine Serum Albumin (BSA) in water in the concentration range of 0.0 - 0.5 mg/mL was used for determination of the standard curve. The assay was started by adding 4 mL of BCA reagent to each of the samples, and to the same volume (500 µL) of the standard solutions, likewise. After 4 h of incubation at room temperature under agitation, the absorption of the supernatants was measured at 562 nm. Plotting of the absorption values of the standards vs. the respective concentration yielded the standard curve to which the data of the resin samples was fitted. The standardized ligand densities are expressed as mg Nanofitin per mL resin.

SLO-Protein Binding Assay (SLO-PB)

To evaluate the ligand immobilization, a binding assay for the SLO target was established. The assay was performed with fixed concentrations of protein and resin, corresponding to a 1.3 - 2 fold excess of SLO protein in relation to the amount of immobilized Nanofitin.

The assay was carried out as follows: 50µL of a 50% slurry (corresponding to 25µL settled resin) was added to Spin-X® centrifuge tube filters with a 0.45 µm cellulose acetate membrane (Corning). After equilibration with 10 mM phosphate buffer, pH 7.2, the mixture was centrifuged at 13200 rpm and the supernatant discarded. 400µL of the SLO target at a concentration of 1 mg/mL was added to the resin and incubated for 2 h under agitation. The absorption of the supernatant at 280 nm was then measured and the PB calculated as follows:

$$PB \left( \frac{mg SLO}{mL resin} \right)= \frac{V_{SLO}\cdot(c_{SLO, preload}- c_{SLO, after 2 h Incubation})}{V_{resin}}= \frac{0.4 mL \cdot(1 \frac{mg}{mL}- c_{Supernatant})}{0.025 mL}$$

Supplementary Table 1: Composition of the stock solutions used in Nanofitin immobilization. All reagents are from Merck Millipore if noted otherwise.

|  | **Substance** | **Concentration** |
| --- | --- | --- |
| **Nanofitins® stock solution (pH 9)** | Purified Nanofitins**® in coupling buffer** | 4 g.L^-1^ |
|  |  |  |
| **Coupling solution (pH 9)** | Sodium Sulphate | 1.4 M |
|  | Potassium dihydrogen phosphate | 25 mM |
|  | Ditiotreitol (DTT) | 30 mM |
|  | Ethylenediamine tetraacetic acid (EDTA) | 2 mM |
|  | | |
| **Deactivation solution (pH 9)** | Glycine | 1 M |
|  | | |
| **Storage solution** | Ethanol | 20% (v/v) |
|  | Sodium chloride | 150 mM |
|  |  |  |

Supplementary Figure 1

**
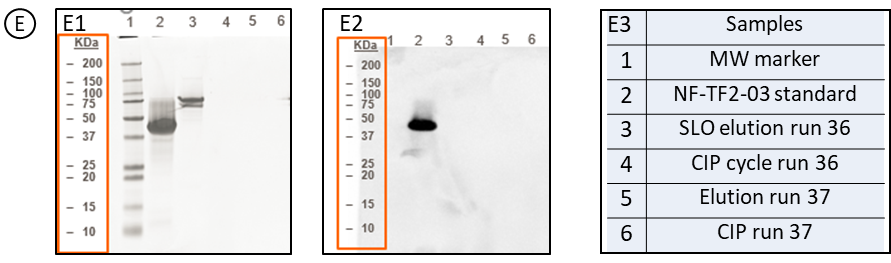

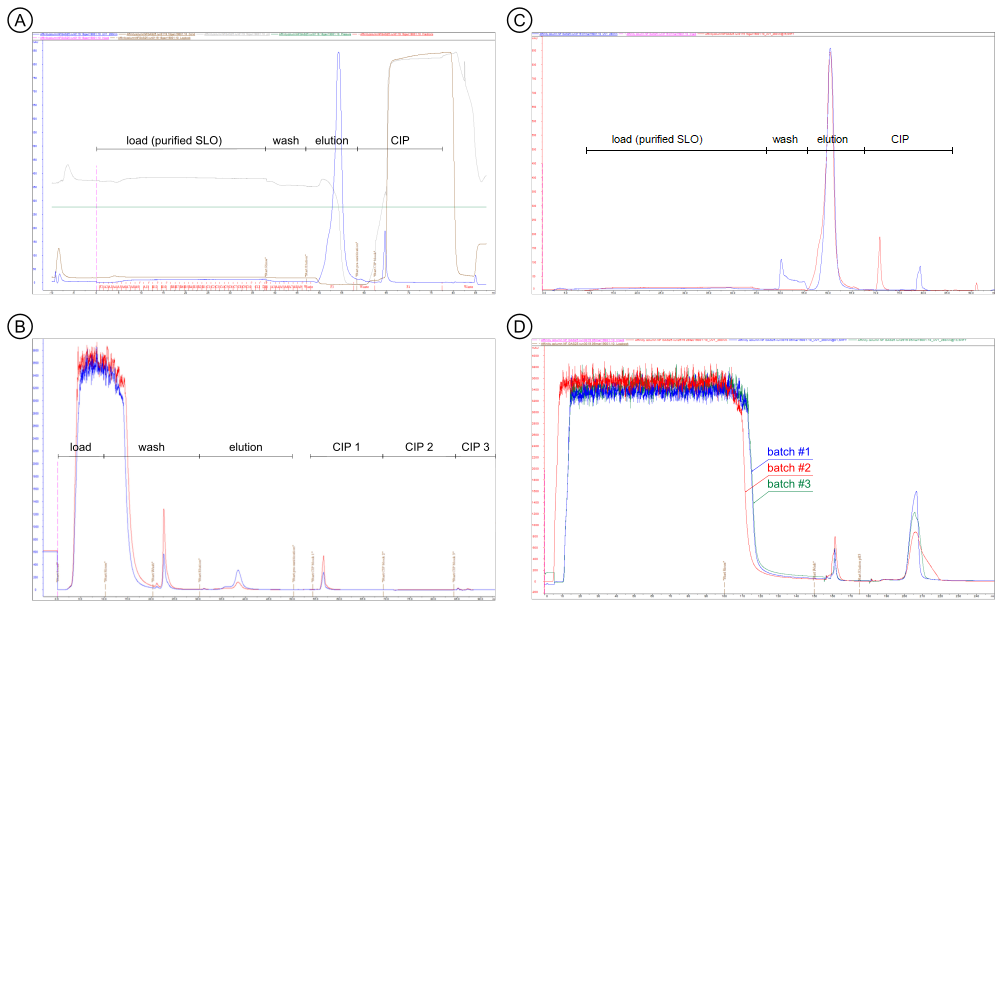
**

**Caption:** Affinity process development on 1 mL column A) Explorative study for dynamic binding capacity determination using 15 mg of pure SLO loaded on the 1 mL column; B) Determination of efficient cleaning step to remove contaminant from resin: after loading crude SLO and performing wash and elution according to develop methods, CIP steps have been realized consecutively at 0.25, 0.5 and 1M of NaOH as CIP solution; C) Cycling study with CIP step performed at 0.25M NaOH during 10 column volume; Pure SLO was loaded to the chromatography medium, eluted and regenerated for 36 cycles; quantity in elution pic compared based on OD280 nm, blue: first cycle, red: 36th cycle; D) Real feed loading studies performed with 3 different feedstock. E) Detection of leached ligand in eluted and CIP fractions after 36 cycles of column use and loading of purified SLO (sample 3 and 4) and run 37 without loading of SLO, NF-TF2-03 standard(2) is GFP fused, expected at a molecular weight of 35 kDa while ligand on resin is expected around 10 Kda in the other samples, SLO protein is expected at 60 kDa, E1: SDS-PAGE of samples, E2: Western Blot revealed by anti-RGSHHH-HRP antibody specific to detect Nanofitin.

Supplementary Figure 2


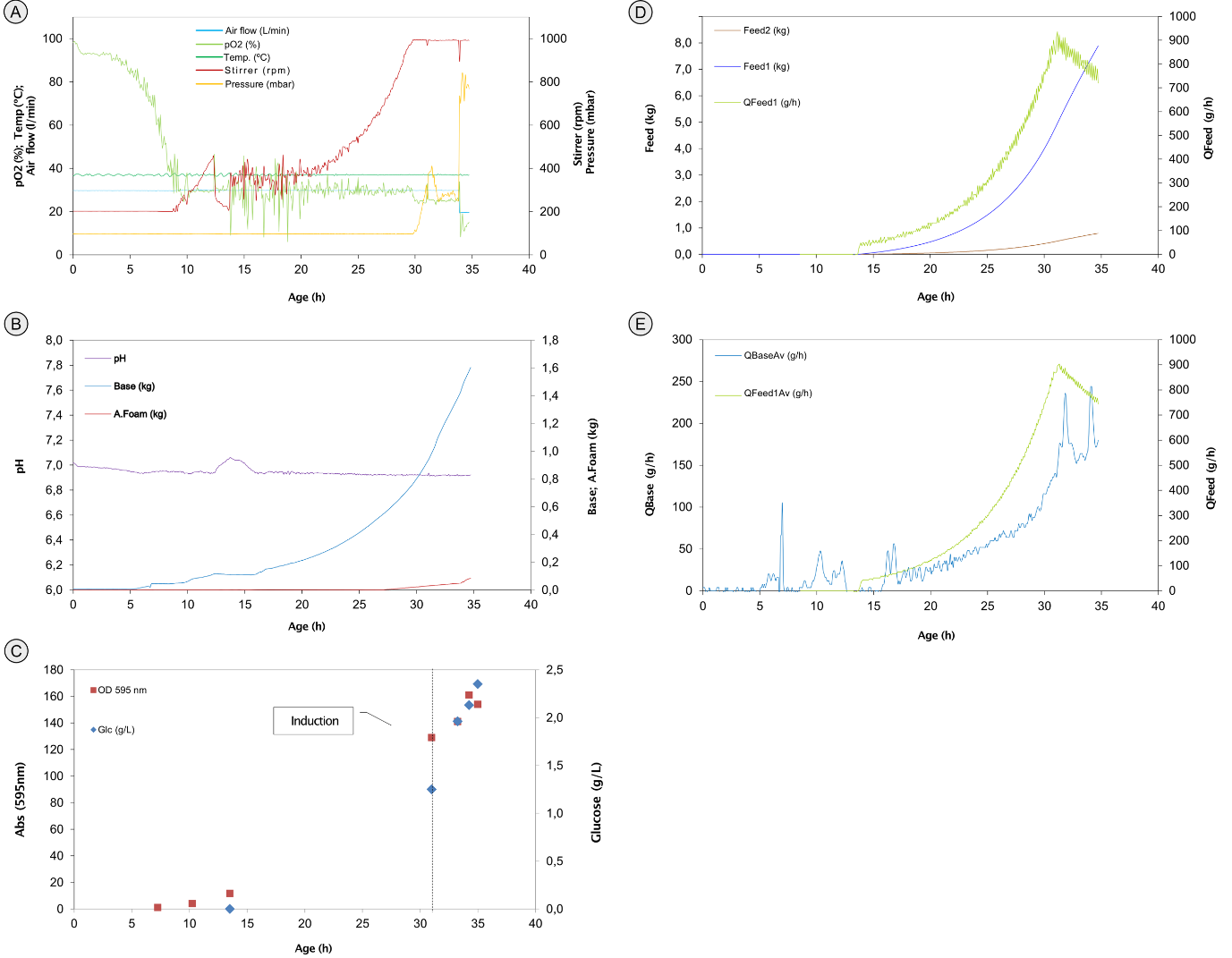


**Caption:** Representative temporal profiles of Nanofitin expression in a fed-batch reaction. A) Air flow, pO2, temperature, stirring speed and pressure; B) pH control; C) Optical density and glucose concentration; D) Feeding profiles; E) averaged feeding profiles for base and Feed1.

Supplementary Figure 3
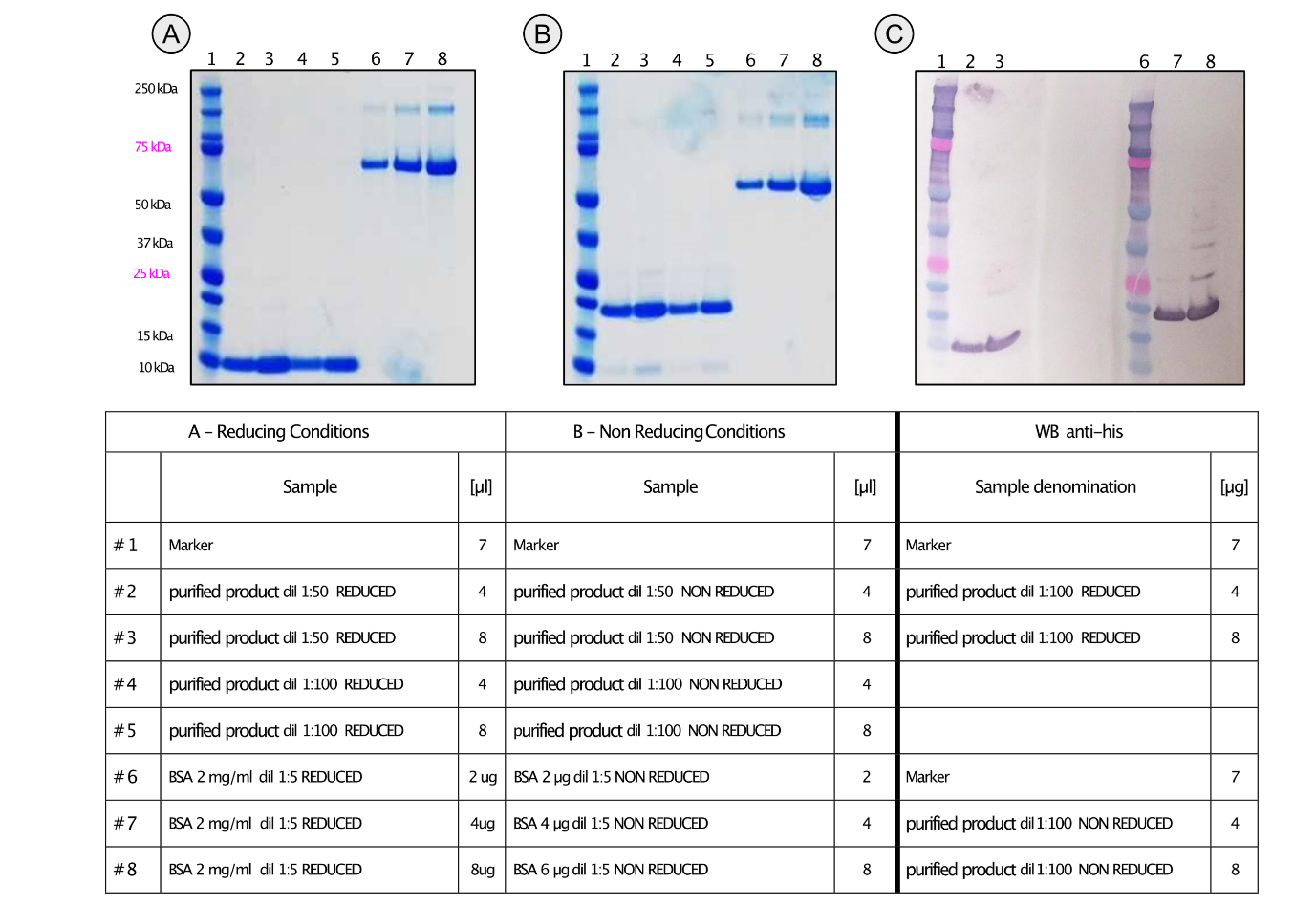


**Caption:** Representative SDS-PAGE and western blot analysis of Nanofitin purified product. A) Reducing conditions;  B) Non-reducing conditions C) western blot analysis using anti-his

Supplementary Figure 4


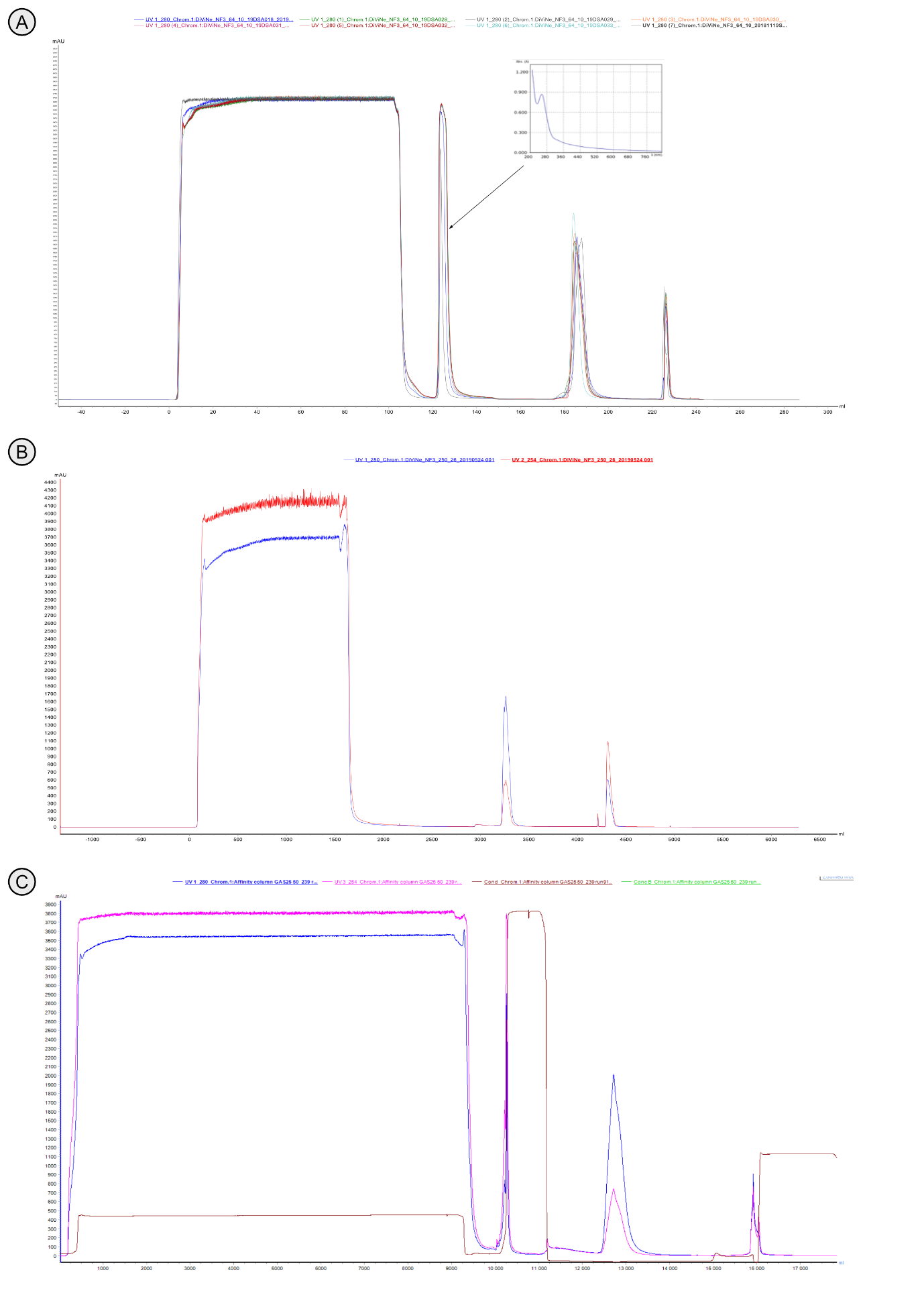


**Caption:** Chromatogram (OD280 nm) depicting the impact of a High salt wash step. The wash peak was later investigated by spectroscopy and confirmed as DNA.
